# Lineage 1 and 2 Strains of Encephalitic West Nile Virus, Central Europe

**DOI:** 10.3201/eid1204.051379

**Published:** 2006-04

**Authors:** Tamás Bakonyi, Éva Ivanics, Károly Erdélyi, Krisztina Ursu, Emőke Ferenczi, Herbert Weissenböck, Norbert Nowotny

**Affiliations:** * University of Veterinary Medicine, Vienna, Austria;; † Szent István University, Budapest, Hungary;; ‡ Central Veterinary Institute, Budapest, Hungary;; § "Béla Johan" National Center for Epidemiology, Budapest, Hungary;; ¶ United Arab Emirates University, Al Ain, United Arab Emirates

**Keywords:** Encephalitis, Arbovirus, West Nile Fever, West Nile virus, Japanese encephalitis virus serogroup, goose, goshawk, emerging infection, research

## Abstract

Two different West Nile virus (WNV) strains caused lethal encephalitis in a flock of geese and a goshawk in southeastern Hungary in 2003 and 2004, respectively. During the outbreak in geese, 14 confirmed human cases of WNV encephalitis and meningitis were reported in the same area. Sequencing of complete genomes of both WNV strains and phylogenetic analyses showed that the goose-derived strain exhibits closest genetic relationship to strains isolated in 1998 in Israel and to the strain that emerged in 1999 in the United States. WNV derived from the goshawk showed the highest identity to WNV strains of lineage 2 isolated in central Africa. The same strain reemerged in 2005 in the same location, which suggests that the virus may have overwintered in Europe. The emergence of an exotic WNV strain in Hungary emphasizes the role of migrating birds in introducing new viruses to Europe.

## Abstract 2

An encephalitic lineage 2 strain of WNV is observed for the first time outside Africa.

Geographically, West Nile virus (WNV) is the most widespread member of the Japanese encephalitis virus (JEV) complex within the genus *Flavivirus* and the family *Flaviviridae*. The first strain (B 956) was isolated from a human patient in the West Nile district of Uganda in 1937; later the virus was also detected in several mosquito species, horses, humans, and other hosts in Africa, Europe, Asia, and Australia (where it has been named Kunjin virus) (*1**–**3*). WNV was introduced into the United States in 1999, and it spread quickly over large parts of North America and reached Mexico (*4**–**7*). The clinical impact of WNV varies in different regions. In the Old World, WNV causes relatively mild infections with influenzalike symptoms or no apparent disease (*2*); encephalitis and fatalities in the human population, horses, or poultry are spasmodic (*3**,**8**,**9*). In the New World, WNV exhibits increased virulence among the local wild bird populations and causes more frequent severe central nervous system symptoms and deaths in humans and horses (*6**,**10*). Although exactly how WNV was introduced into New York is unclear, phylogenetic comparison of the viral nucleic acid sequences has shown a close relationship between the American WNV isolates and strains isolated from encephalitic geese and storks in Israel in 1998 (*11**–**13*). Experimental infections of rodents indicated that the neurovirulence of WNV correlates with its genotype, and the North American strains are highly neurovirulent for mice (*14*).

WNV shows relatively high levels of sequence diversity. Comprehensive studies on the phylogenetic relatedness of WNV strains show that they form at least 2 main lineages (*15**–**17*). Lineage 1 is composed of WNV strains from different geographic regions, and it is subdivided into at least 3 clades. Clade A contains strains from Europe, Africa, the Middle East, and America; clade B represents the Australian (Kunjin) strains; and clade C contains Indian WNV isolates. Lineage 2 contains the B 956 prototype strain and other strains isolated so far exclusively in sub-Saharan Africa and Madagascar. In addition to the 2 major WNV lineages, we recently proposed 2 lineages for viruses that exhibited considerable genetic differences to the known WNV lineages: lineage 3 consists of a virus strain isolated from *Culex pipiens* mosquitoes at the Czech Republic/Austria border (named Rabensburg virus), and lineage 4 consists of a unique virus isolated in the Caucasus. These 2 viruses, however, may also be considered independent flaviviruses within the JEV complex (*18*).

WNV has been known to be present in central Europe for a long time. Seroprevalence in humans was reported in several countries, including Hungary, and WNV strains were isolated from mosquitoes, humans, migrating birds, and rodents during the last 30 years (*3*). Until 2003, however, WNV infections in Hungary have never been associated with clinical symptoms, although a severe outbreak of West Nile encephalitis in humans was reported in 1996 and 1997 in neighboring Romania.

In late summer 2003, an outbreak of encephalitis emerged in a Hungarian goose flock, resulting in a 14% death rate among 6-week-old geese (*Anser anser domesticus*). Based on histopathologic alterations, serologic investigations, and nucleic acid detection by reverse transcription–polymerase chain reaction (RT-PCR), WNV was diagnosed as the cause of the disease (*19*). Chronologically and geographically related to the outbreak in geese, a serologically confirmed WNV outbreak was also observed in humans, which involved 14 cases of mild encephalitis and meningitis (*20*).

One year later, in August 2004, a goshawk (*Accipiter gentilis*) fledgling showed central nervous system symptoms and died in a national park in southeastern Hungary. When histopathologic methods and RT-PCR were used, WNV antigen and nucleic acid were detected in the organs of the bird. Furthermore, the virus was isolated after injection of suckling mice. Here we report the sequencing and phylogenetic results of these 2 encephalitic WNV strains that emerged recently in central Europe.

## Materials and Methods

Brain specimens from one 6-week-old goose, which died during the encephalitis outbreak in a Hungarian goose flock, and brain samples from a goshawk, which also died from encephalitis, were used for WNV nucleic acid determination. The brain samples were homogenized in ceramic mortars by using sterile quartz sand, and the homogenates were suspended in RNase-free distilled water. Samples were stored at –80°C until nucleic acid extraction was performed.

Viral RNA was extracted from 140 μL of brain homogenates by using the QIAamp viral RNA Mini Kit (Qiagen, Hilden, Germany) according to the manufacturer's instructions. First, a universal JEV-group specific oligonucleotide primer pair designed on the nonstructural protein 5 (NS5) and 3´-untranslated regions (UTR) of WNV (forward primer: 5´-GARTGGATGACVACRGAAGACATGCT-3´ and reverse primer: 5´-GGGGTCTCCTCTAACCTCTAGTCCTT-3´ [21]; ) was applied on the RNA extracts in a continuous RT-PCR system employing the QIAGEN OneStep RT-PCR Kit (Qiagen). Each 25-μL reaction mixture contained 5 μL of 5× buffer (final MgCl_2_ concentration 2.5 mmol/L), 0.4 mmol/L of each deoxynucleoside triphosphate, 10 U RNasin RNase Inhibitor (Promega, Madison, WI, USA), 20 pmol of the genomic and reverse primers, 1 μL enzyme mix (containing Omniscript and Sensiscript Reverse Transcriptases and HotStarTaq DNA polymerase) and 2.5 μL template RNA. Reverse transcription was carried out at 50°C for 30 min, followed by a denaturation step at 95°C for 15 min. Thereafter, the cDNA was amplified in 40 cycles of heat denaturation at 94°C for 40 s, primer annealing at 57°C for 50 s, and DNA extension at 72°C for 1 min, and the reaction was completed by a final extension for 7 min at 72°C. Reactions were performed in a Perkin-Elmer GeneAmp PCR System 2400 thermocycler (Wellesley, MA, USA) and in a Hybaid PCR Sprint thermocycler (Thermo Electron Corporation, Waltham, MA, USA).

After RT-PCR, 10 μL of the amplicons was subjected to electrophoresis in a 1.2% Tris acetate-EDTA-agarose gel at 5 V/cm for 80 min. The gel was stained with ethidium bromide; bands were visualized under UV light and photographed with a Kodak DS Electrophoresis Documentation and Analysis System using the Kodak Digital Science 1D software program (Eastman Kodak Company, Rochester, NY, USA). Product sizes were determined with reference to a 100-bp DNA ladder (Promega).

Where clear PCR products of the previously calculated sizes were observed, the fragments were excised from the gel, and DNA was extracted by using the QIAquick Gel Extraction Kit (Qiagen). Fluorescence-based direct sequencing was performed in both directions on PCR products. Sequencing of PCR products was carried out with the ABI Prism Big Dye Terminator cycle sequencing ready reaction kit (Perkin-Elmer), according to the manufacturer's instructions, and an ABI Prism 310 genetic analyzer (Perkin-Elmer) automated sequencing system. Nucleotide sequences were identified by Basic Local Alignment Search Tool (BLAST, http://www.ncbi.nlm.nih.gov/blast) search against gene bank databases. Based on the sequence information obtained from the amplification products, complete WNV sequences that exhibited the highest nucleotide identities with the Hungarian genotypes were selected from the GenBank database to design primers that amplify overlapping RT-PCR products covering the entire genome of the strains. Oligonucleotide primers were designed with the help of the Primer Designer 4 for Windows 95 (Scientific and Educational Software, Version 4.10; Microsoft, Redmond, WA, USA) and were synthesized by GibcoBRL Life Technologies, Ltd. (Paisley, Scotland, UK). Detailed information on all primers is in the Tables A1 and A2. PCR amplification products were directly sequenced in both directions; the sequences were compiled and aligned to complete genome sequences of selected representatives of WNV lineages 1a, 1b, 2, and putative lineages 3 and 4 (listed in Table). Phylogenetic analysis was performed by using the modified neighbor-joining method (ClustalX [22]; ), and trees were constructed to demonstrate the relationship between the Hungarian WNVs and other WNV strains (Figure).

**Figure:**
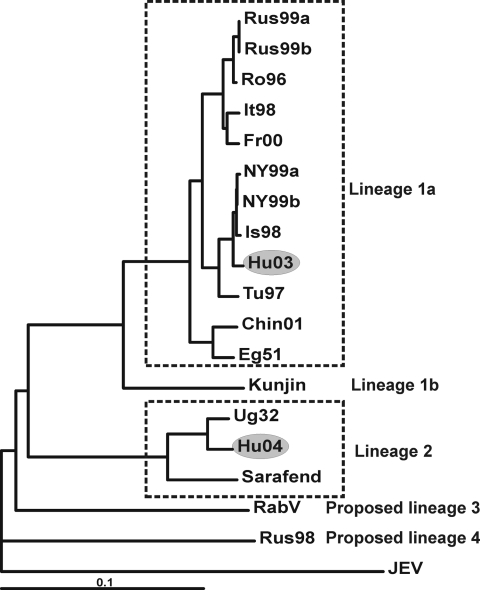
Phylogenetic tree based on the complete nucleotide sequences of selected West Nile virus strains demonstrating the genetic relatedness of these strains (abbreviations are listed in Table). Boxes indicate different lineages and clades. The Hungarian strains reported in this article are highlighted with gray background). RabV, Rabensburg virus; JEV, Japanese encephalitis virus. Scale bar depicts degree of relatedness.

**Table:** West Nile virus strains included in the phylogenetic analysis

Name	Code	Accession no.	Isolation			
			Year	Host	Origin	Lineage, clade
WNV HNY1999	NY99a	AF202541	1999	Human	New York, USA	1a
WNV NY99flamingo38299	NY99b	AF196835	1999	Flamingo	New York, USA	1a
WNV IS98STD	Is98	AF481864	1998	Stork	Israel	1a
WNV goose-Hungary/03	Hu03	DQ118127	2003	Goose	Hungary	1a
WNV Italy1998Equine	It98	AF404757	1998	Horse	Italy	1a
WNV RO9750	Ro96	AF260969	1996	*Culex pipiens*	Romania	1a
WNV VLG4	Rus99a	AF317203	1999	Human	Volgograd, Russia	1a
WNV LEIV-Vlg99-27889	Rus99b	AY277252	1999	Human	Volgograd, Russia	1a
WNV PaH001	Tu97	AY268133	1997	Human	Tunisia	1a
WNV PaAn001	Fr00	AY268132	2000	Horse	France	1a
WNV Eg 101	Eg51	AF260968	1951	Human	Egypt	1a
WNV Chin-01	Chin01	AY490240	1950s	?	Russia	1a
WNV Kunjin MRM61C	Kunjin	D00246	1960	*Cx. annulirostris*	Australia	1b
WNV Sarafend	Sarafend	AY688948	Laboratory strain			2
WNV B956 (WNFCG)	Ug37	NC_001563	1937	Human	Uganda	2
WNV goshawk-Hungary/04	Hu04	DQ116961	2004	Goshawk	Hungary	2
Rabensburg virus (97-103)	RabV	AY765264	1997	*Cx. pipiens*	Czech R.	3?
WNV LEIV-Krnd88-190	Rus98	AY277251	1998	*Dermacentor marginatus*	Caucasus, Russia (Georgia?)	4?

The nucleotide sequences of the Hungarian WNV strains goose-Hungary/03 (Hu03) and goshawk-Hungary/04 (Hu04) were submitted to the GenBank database. They are available under accession numbers DQ118127 and DQ116961, respectively.

## Results

In this study, the complete genome sequences of WNV strains derived from a 6-week-old goose, which died in 2003 during an outbreak of encephalitis in a Hungarian goose flock (strain goose-Hungary/03), and from a goshawk, which also died from encephalitis in the same region 1 year later (strain goshawk-Hungary/04), were determined, aligned, and phylogenetically analyzed. The genome of the goose-Hungary/03 strain is composed of 10,969 nucleotides (nt) and contains 1 open reading frame between nucleotide positions 97 and 10,398, coding for a 3,433 amino acid (aa)–long putative polyprotein precursor. The complete genomic sequence of the virus was subjected to a BLAST search against gene bank databases. The highest identity rates (98% at the nucleotide and 99% at the amino acid level) were found with WNV strains isolated in 1998 in Israel and in 1999 in the United States. In addition, phylogenetic analysis was performed to indicate the relationships between the Hungarian goose–derived WNV strain and selected representatives of WNV clades and clusters. The resulting phylogenetic tree (Figure) confirmed the results of the BLAST search, i.e., the Hungarian goose–derived WNV strain is clustering close to the previously mentioned WNV strains isolated in the United States and Israel, which belong to lineage 1a of WNV. Other European WNV strains (isolated in Italy, France, and Romania) are more distant to the Hungarian strain; they form a separate cluster consisting of a Romanian/Russian and a French/Italian subcluster.

The complete nucleotide sequence of the goshawk-Hungary/04 WNV strain is composed of 11,028 nt and contains 1 open reading frame between nucleotide positions 97 and 10,401, coding for a 3,434-aa putative polyprotein precursor. In BLAST search, the strain showed the highest (96% nt and 99% aa) identity to the WNV prototype strain B 956. Consequently, as the phylogram also indicates (Figure), this virus belongs to lineage 2 of WNV. Alignments of the available partial sequences from the E protein coding regions of other representatives of this cluster showed even higher identities (97%–98% nt and 100% aa) with WNV strains isolated in central Africa in 1972 (AnB3507, AF001563) and in 1983 (HB83P55, AF001557), respectively (*15*).

More recently (in early August 2005), additional lethal cases of encephalitis occurred in birds of prey in the same place in which the goshawk died of West Nile encephalitis in 2004, involving up to a total of 3 goshawks and 2 sparrow hawks (*A. nisus*); 2 of the goshawks and 1 sparrow hawk died. Preliminary investigations detected WNV-specific nucleic acid in the brains of the birds. The partial nucleotide sequence of the 2005 virus (1,000 bp at the NS5´–3´-UTR regions) showed 99.9% identity with the goshawk-Hungary/04 strain (only 1 substitution at nucleotide position 9,376 [g→a] has been observed, which did not influence the putative amino acid sequence). Additional observation of the outbreak and investigations of the cases are in progress.

## Discussion

The primary aim of our investigations was to show the genetic relatedness of the WNV strains detected in Hungary in the last 2 years and to estimate their clinical and epidemiologic impact. The phylogenetic analysis emphasizes the close genetic relationship of the goose-Hungary/03 strain with a WNV strain isolated in Israel in 1998 and the WNV strain introduced in New York in 1999, since the 3 WNVs form 1 single cluster within clade 1a of lineage 1. These strains caused outbreaks in birds, humans, and horses. Previous European WNV isolates exhibited lower identity values, e.g., the strain that was responsible for the Romanian outbreak(s) in 1996 and 1997 showed only 96% nt identity with the Hungarian goose-2003 strain, and in the phylogenetic tree the other European isolates form a separate cluster consisting of 2 subclusters (Figure). The earliest representatives of the Israel/USA/goose-Hungary/03 cluster were reported by Malkinson et al. (*23*) from ill and dead white storks (*Ciconia ciconia*) in Israel in 1998. These storks, however, had hatched in central Europe, and during their autumn migration southwards, strong winds had blown them off course, from their usual route to Africa, to southern Israel. Malkinson et al. suspected that these birds introduced the neurovirulent genotype of WNV to Israel from their hatching place. The wetlands of southeastern Hungary are foraging and nesting habitats for storks and many other wild bird species, and the goose farm, where the WNV outbreak occurred in 2003, is located in this region. These facts, together with the close phylogenetic relatedness of the Israeli/US/Hungarian WNV strains, strongly support the theory that storks carried the neurovirulent WNV strain from central Europe (that is, from Hungary) to Israel, which sheds new light on the introduction of WNV to New York. This virus could have originated in Israel (which is the generally accepted although not proven theory) or central Europe. In both cases, however, the virus seems to have its true origin in Europe. In a recent publication, Lvov et al. suggested that WNV could have been introduced into New York by ships traveling from Black Sea ports (*24*).

When a WNV infection was detected in 2004 in a goshawk fledgling, which died from encephalitis in the same region of Hungary in which the outbreak in geese and humans occurred during the previous year, we anticipated a WNV strain more or less identical to the genotype detected there in 2003. The genomic sequence of this strain was not closely related to the sequence of the WNV strain detected in geese in the year before, however, but belonged to the group of central African lineage 2 WNV strains. A closely related strain from this cluster (ArB3573, AF001565, and AF458349) was identified as a neuroinvasive strain of WNV in a mouse model (*14*). To our knowledge, this report is the first on the emergence of a lineage 2 WNV strain outside Africa. Migratory birds that had overwintered in central Africa probably introduced this exotic strain to the wetlands of Hungary. On the other hand, as the goshawk is not a migratory species, and infection occurred in August, the African WNV strain must have already successfully adapted to local mosquito vectors. Consequently, this neurotropic, exotic WNV strain may become a resident pathogen in Europe with all the possible public health consequences.

Our results indicate that the WNV strains that emerged in 2 consecutive years and caused avian deaths in Hungary are epidemiologically unrelated. Genetically distinct WNV strains are circulating simultaneously yet independently in local birds and thus most likely also in local mosquito populations within the same region. They cause sporadic cases of encephalitis and also raise the possibility of spreading to other European countries or even to other continents, as happened in 1999 with another WNV strain, which resulted in a public health catastrophe in America.

In addition to the above 2 novel WNVs, we recently characterized another novel flavivirus of so far unknown human pathogenicity named Rabensburg virus, which has been isolated from *Culex pipiens* mosquitoes in 1997 and 1999 at the Czech Republic–Austria border, only a few hundred kilometers from the region where the Hungarian WNVs emerged. After the entire genome was sequenced, Rabensburg virus turned out to represent either a new (third) lineage of WNV or a novel flavivirus of the JEV group (*18*). Thus, several distinct WNV strains seem to circulate in central Europe. In 2001 another flavivirus of the JEV group, Usutu virus, which has never previously been observed outside Africa, emerged in Austria and resulted in deaths in several species of birds, especially Eurasian blackbirds (*Turdus merula*) (*21*). This virus became a resident pathogen in Austria and continues to disperse and cause deaths in blackbirds and other species of birds (*25**,**26*).

The snowy winter and rainy spring of 2005 resulted in serious floods in the area in which the Hungarian WNV strains were identified. Since the floodplains and polders were under water, the conditions for mosquito development were ideal. The summer was also very rainy, which resulted in more floods in the region and continuous mosquito gradation. The most recent data imply that the lineage 2 WNV strain may have overwintered in Hungary, causing several clinical cases of encephalitis in *Accipiter* species in 2005 as well.

The routine diagnostic techniques in most of the European public health and veterinary laboratories are designed to detect lineage 1 WNV strains. In a recent PCR external quality assurance multicenter test, <40% of the involved laboratories could detect lineage 2 WNV strains (Matthias Niedrig, pers. comm.). Therefore, a major goal of this article is to increase the scientific and public awareness of this potential public health threat for Europe and, perhaps, America. Furthermore, comprehensive investigations on the occurrence, ecology, and epidemiology of the different WNV strains circulating in central Europe, as well as the development of monitoring and surveillance programs, must be of highest priority. One may also speculate on environmental factors, such as climate change or global warming, that may have enhanced the recent emergence of viruses, which had previously been restricted to Africa, in new habitats and continents. Improved observation, reporting, and detection methods have also contributed to the apparent increasing emergence of these viruses.

## Appendix

**Table A1:** Oligonucleotide primers used for sequencing West Nile virus (WNV) strain Goose-Hungary/03*

Name*	Sequence 5´ to 3´	Product length (bp)
WNVI 1f	AGTAGTTCGCCTGTGTGAGC	388
WNVI 388r	GCCGATTGATAGCACTGGTC	
WNVI 82f	AGCACGAAGATCTCGATGTC	986
WNVI 1067r	ATGATAGTCACGCAGCTGTC	
WNVI 1005f	AGGAGTGTCTGGAGCAACAT	995
WNVI 1999r	AAGCCACTGACGAGATAGGA	
WNVI 1864f	ACAACCTATGGCGTCTGTTC	1,049
WNVI 2912r	CGATTCTGAGTCGGACATTC	
WNVI 2847f	AGAACTCGCCAACAACACCT	774
WNVI 3620r	ATCTTGGCTGTCCACCTCTT	
WNVI 3502f	CTCGTGCAGTCACAAGTGAA	957
WNVI 4458r	ATCAAGCCGCACATCAACTC	
WNVI 4524f	GGTCTGTCTCGCGATTAGTG	500
WNVI 5023r	GTGAGCCTGATGTTCCAGTG	
WNVI 5300f	CTGAGATGGCTGAAGCACTG	858
WNVI 6157r	TCTCACGCTCTGGTTGGTAG	
WNVI 5303f	AGATGGCTGAAGCACTGAGA	162
WNVI 5464r	CCATCACGAACAGGTTGTAG	
WNVI 6009f	GTCGCAAGTTGGTGATGAGT	228
WNVI 6236r	TCTGCAGTCCTCAACAGTTC	
WNVI 6119f	ACGGACTGATCGCTCAATTC	1,151
WNVI 7269r	AAGGAGTGTTGCCGCTGTTA	
WNVI 7177f	TTCGTCGATGTTGGAGTGTC	993
WNVI 8169r	CCGAATCGTCCTATGCTCTT	
WNVI 8112f	TTGTGACATCGGAGAGTCCT	1068
WNVI 9179r	AGCCAGTGGTCTTCATTGAG	
WNVI 8950f	GCCAGAGAAGCAGTTGAAGA	305
WNVI 9254r	CCAACTTCACGCAGGATGTA	
WNVI 9134f	TTCTGGAGTTCGAGGCTCTG	1,186
WNVI 10319r	CCGATGATTGCTCTGACTTG	
WNVI 9445f	GGAAGAACCGTCATGGATGT	466
WNVI 9910r	GAGCTCTGCCTACCAATTCA	
WNVI 10524f	CGCCACCGGAAGTTGAGTAG	501
WNVI 11024r	CTGTGTTCTCGCACCACCAG	

*Numbers refer to nucleic acid positions at the 5´-end of the primers, according to the complete genomic sequence of the closely related WNV strain WN NY 2000-crow3356 (AF404756 27; ). f, forward (genomic) primer; r, reverse (complementary) primer.

**Table A2:** Oligonucleotide primers used for sequencing of West Nile virus (WNV) strain goshawk-Hungary/04

Name*	Sequence 5´ to 3´	Product length (bp)
WNVII 1f	AGTAGTTCGCCTGTGTGAGC	200
WNVII 200r	AGCATAGCCCTCTTCAGTCC	
WNVII 81f	TGGCACGAAGATCTCGATGT	874
WNVII 954r	TGCCACCAGGAGCAATAGAA	
WNVII 870f	CCTCGTTGCAGCTGTCATTG	761
WNVII 1630r	TCCATGGCAGGTTCAGATCC	
WNVII 1584f	CTTCCTGGTTCACCGAGAAT	908
WNVII 2491r	CTTGCCTGCCAATGTCAATG	
*Usu 2328f*	CTCTTTGGRGGRATGTCHTG	818
*Usu 3145r*	GTGCADGATTTKACYTCTCC	
WNVII 3027f	CAACATGGCTGTGCATAGTG	673
WNVII 3699r	GCCGACGAGAATGACATATC	
*Usu 3606f*	AAGAGGTGGACGGCCARRHT	1,152
*Usu 4759r*	GTGTGCCAYAGYGTGTGGAA	
WNVII 3861f	GGCTTACTATGACGCCAAGA	594
WNVII 4454r	CCATCATCATCCAGCCTAAC	
*Usu 4251f*	ATGTTYGCCATYGTWGGDGG	1,142
*Usu 5392r*	TGGCACATSACATCMACKAT	
WNVII 5294f	TTGCTGCTGAGATGTCTGAG	324
WNVII 5617r	TGTCCGAGATAGGAGCATTG	
*Usu 5454f*	ATGGATGAAGCYCATTTCAC	337
*Usu 5790r*	GGTAYTCYGTMTCATAGGAC	
WNVII 5629f	GCCTGGAACACTGGATATGA	616
WNVII 6245r	TAAGCGAGCCAGACTGGTAA	
WNVII 6127f	TATCAGCCTGAGCGCGAGAA	932
WNVII 7058r	ACGGCTGTCGTTACGGCATA	
WNVII 6935f	ATGACATTGGCAGCCTGTTG	672
WNVII 7606r	ATCCTCCTCGCATGATGTGA	
WNVII 7505f	GAGAGGCTGGAATTCTGACT	653
WNVII 8158r	GTTCTTCTACCTCGGCACTT	
WNV 8078f	CATCAGAAGCGAGCGACACA	744
WNV 8821r	ACAGCCAGTTCGTGGTCTCA	
WNV 8723f	TCGGTCAACAACGAGTGTTC	739
WNV 9461r	GAGATGACGTCCATCACAGT	
WNV 9368f	TAGCGCGGTCCATCATCGAG	733
WNV 10100r	CCAACGTTCGACCGGAGTCT	
WNVII 10023f	CTGTTCCGCTGTGCCTGTCA	519
WNVII 10541r	CGTGGATCACCTCGCAGCTT	
*WNV 9031f*	TACAACATGATGGGVAARAGAGAGA	1,061
*WNV 10091r*	AGCATGTCTTCYGTBGTCATCCAYT	
WNV 10460f	GCCACCGGAAGTTGAGTAGA	430
WNV 10889r	GCTGGTTGTGCAGAGCAGAA	
*Usu 10596f*	GWAAGCCTCYCAGAACCGTCTCGGAAG	419
*Usu 11014r*	AGATCCTGTGKTCTWSYYCMCCAYCAG	

*Numbers refer to nucleic acid positions at the 5´-end, according to the complete genomic sequence of the closely related lineage 2 WNV strain B956 (NC_001563). Universal, Japanese encephalitis virus–group specific primers are indicated in *italics* (*28*). f, forward (genomic) primer; r, reverse (complementary) primer. Usu, Usutu.
